# The Rites of Passage Framework as a Matrix of Transgression Processes in the Life Course

**DOI:** 10.1007/s10804-018-9285-1

**Published:** 2018-01-22

**Authors:** Bernadetta Janusz, Maciej Walkiewicz

**Affiliations:** 1 Faculty of Psychiatry, Jagiellonian University in Kraków, Collegium Medicum, Kopernika 21a Street, 31-501 Kraków, Poland; 2 Faculty of Psychology, Medical University of Gdańsk, Tuwima 15 Street, 80-210 Gdańsk, Poland

**Keywords:** Rite of passage, Life course, Health crisis, Turning point, Transgressive processes, Functional–structural analysis

## Abstract

This work shows the contribution of concept of rites of passage and theory of liminality to the understanding of transformations in the course of a person’s life. The structural–functional analysis of empirical studies of physical changes, changing roles in society, and key changes in the area of mental and physical health conducted from the perspective of these theories has allowed to identify the three fundamental processes that govern the attainment of transformation and transgression into a new phase of life. The aim of this paper is to set out the processes identified in the course of functional–structural analysis of chosen studies and they comprise: (1) preservation of the sequence of the life course; (2) liminality: deconstruction, integration, and transformation; and (3) performativity. These processes provide a structural framework for understanding life crises, thus facilitating their study as phases of dynamic transformations connected with the successive roles and tasks over the life course.

## Background

Arnold van Gennep’s concept of the rite of passage (Cheal 1988; Van Gennep 1960) was developed with the aim of systematizing the rituals and rites connected with change in the status of the individual in homogeneous primitive societies. In this paper, we want to show that it is still a significant theoretical and research construct. This theory cannot be directly applied to description of rituals and rites in contemporary developed societies. Contemporary human life unfolds in conditions of increasing space for individual choices, the breakdown of local communities, and diversification of life options (Frank and Meyer 2002; Kumar 2004). We nevertheless support the belief that, irrespective of whether they live in traditional or more complex societies, people are constantly confronted with fundamental changes in their lives (Cheal 1988), and that modernity is more a ‘multiplicity of patterns and ways of life’ than a fundamental difference in them (Herzfeld 2000). We thus hold that the concept of the rite of passage continues to offer a dynamic, processual structure that enables us to study transitions within life cycles and the institutions related to them (Froggatt 1997).

In this work, we refer to the concept of the rite of passage, to the concept of the life course that are directly connected with changes and life transformations, key life trajectories, and the sequence of successive life-changing events (Giele and Elder 1998; Shanahan and Elder 2002). Both concepts describe changes both in an individual’s internal world and, parallelly, in the social world. Both are based in the temporal perspective, and both relate to two opposing dimensions of human life—continuity and change, i.e., transformation.

The life course concept introduces the key life-trajectories like personal, occupational, biological ones with its main transition points like the birth of a child, a new job, growing up with all possible consequences of temporal, sequential and other disruptions while going through these phases. At the same time, van Gennep’s concept refers to similar life transitions that are accompanied by rites of passage. What is more, the structure of rite of passage shows possible dangers of disruptions in the sequence of life events connected with certain social roles.

## Method

In this paper, van Gennep’s concept of the rite of passage (Van Gennep 1960) and Turner’s theory of liminality (Turner 1967) are treated as a theoretical matrix enabling us to analyze transitional periods in the life course from structural–functional perspective and the ways in which they change in different cultures and times, e.g., how the passage through the period of mourning is shortened (Littlewood 1993) and the accession to adulthood as a separate, self-sufficient life is lengthened, differentiated (Kins and Beyers 2010) or blurred (Settersten et al. 2005; Twenge 2008).

In order to present our concept of functional–structural analysis, we want to point out that three-stage sequence established by Van Gennep (1960), that is, separation, transition, and incorporation, introduces the structural perspective, but its function is creating clearly marked boundaries and establishing social identities and roles. Bell (1997) pointed at the purposes of rite of passage seen from neo-functional perspective as having cathartic functions, affecting cognitive and neurophysiological processes within the body and alleviating anxiety. Turner (1967) puts greater emphasis on the liminal period as a phase when conflicts are dramatized and dominant social values that hold the group together are transmitted, which introduces again the function of the performance. Structural–functional analysis applied in this paper, on its functional level, refers mainly to the function of rites of passage in primary societies and such social structures in contemporaneity that may have similar functions. When referring to structural dimensions of rites of passage and theory of liminality, we used the term of structural matrix as this unique structure facilitates the understanding of a change and transformation in critical situations in the life course. This is possible due to a number of aspects of this theory, including firstly the normative rules surrounding the sequence of societally defined life transitions, secondly the performative aspects of ritual performance, and thirdly the three-level structure it applies to the process of ritual change (Van Gennep 1960). The first, ‘separation phase’ makes symbolic reference to loss, to leaving the previous state, or loss of a position achieved in life. The middle, ‘liminal phase’ is the crisis phase, characterized by uncertainty, disruption of order, and suspension of the structure of society. The third phase is the ‘incorporation phase,’ entailing a new social identity and marking the start of a new period of life (Van Gennep 1960). Another strength of this conception, aside from the modal aspects listed here, is its simultaneous reference to several levels of change: from the level of the body (Peters 1994) to the level of societal transformation (Draper 2003), without compromise to the continuity of the individual identity, or to the continuity of the family or social group (Draper 2003; Turner 1967; Van Gennep 1960). Referring to the structural dimensions of the theory rites of passage and liminality presented above, we have created the term of structural matrix that comprises mutually intertwined main elements of ritual performance and structurally analogous characteristics of mental crisis that are connected with the main passages in the course of life acknowledging cultural norms and values that are involved in.

We have conducted the analysis regarding three groups of theoretical research studies from the structural point of view meaning the collapse of the previous life structure, transformations to an individual’s ego, implying functional perspective, and we investigated ways of overcoming life crises. One example of this is a study of the experience of cancer that takes the patient into a new state of uncertainty and unpredictability that is sometimes compared with the liminal phase (Halliday et al. 2014), or into disengagement from an established rhythm of life and entrance into a liminal state when experiencing dying and mourning (Froggatt 1997; Hockey 2002; Littlewood 1993) that have introduced the structural collapse of previous state.

Therapeutic help itself in situations of crisis also refers to the cessation of previous ways of adaptation and existing in a state of maladaptation until help in creating new functioning mechanisms is forthcoming. Thus, therapeutic work in a crisis is referenced directly to the three-phase structure of the rite of passage (Laird 1988; Wozniak 2009) that enables to see how functional dimensions are interconnected with structural transformations.

The core of this work is structural–functional analysis of empirical studies and some theoretical works, selected according to a number of criteria. Firstly, they were connected with changes in the body (puberty, illness) and the accompanying changes in the realm of the psyche with further consequences over the life course. Secondly, they have dealt with the changes that have a fundamental effect on the social status of an individual and constitute a turning point or watershed in a person’s life. Thirdly, in the theoretical perspective, they adopt directly or indirectly references to the concept of the rite of passage and the theory of liminality derived from it, or the concept of the human life course. This analysis threw up the three main distinct processes that describe the basic conditions for both transformations to be effected and crises in the human life course to be overcome.

Each of those three distinguished processes has structural and functional elements that are present in symbolic and performative way in the rite of passage concept and the theory of liminality as well as in mental crises that are universal for the life course passages. We have distinguished more structural and more functional properties of the rite of passage and liminality theory regarding the health and mental crises although they are both strictly intertwined (Table 1).

**Table 1:** Structural and functional elements of distinguished process of the rite of passage and theory of liminality

Structure	Function
1. *Preservation of the temporal life course sequence* disruption of an established sequence connected with mental crisis, psychopathology, life misfortunes	1. Maintenance of equilibrium in the face of changes
*2. Liminality: deconstruction, integration, transformation* (between dying of the old and the birth of the new structure)	2. Convergence of the opposites: individual and social, biological and cultural, comprising of the conflictual tendencies within oneself
*3. Performativity* clearly defined sequentially of events, multimodal dramatism of the ritual	3. Regulation and creation: accommodating both elements of arising anxiety and ways of overcoming it

### Preservation of the Temporal Life Course Sequence

This process represents the recognition that certain events in the life course should ensue in a certain order and within certain periods of time. This is illustrated by the group of rites of passage that are ‘corrective’ in respect of disrupted sequences of life experiences. It is reflected in empirical studies which show that disruption of an established sequence of life experiences causes mental crisis, psychopathology, and other life misfortunes. Such sequential aspects of life experiences are clearly highlighted in both the theory of rites of passage and the concept of the life course. Just as the rite of passage divided human life into periods and encapsulated the entire process in a ‘mythical ritual scenario’ (Bajburin 1993; Van Gennep 1960), so the life course concept describes the life of man from birth to death from the perspective of its main changes and transformations (Giele and Elder 1998). This concept also comprises a detailed sequence of the socially defined events and roles enacted by the individual in the course of his or her life (Shanahan and Elder 2002). While the rite of passage provided protection from disruption of life course experiences (Levi-Strauss 1962), the life course concept provides a conceptual framework for study of disruptions to life experiences (George 2014; Hogan 1978; Jackson 2004; Pearlin et al. 2005; Van Gundy and Rebellon 2010; Wrosh and Freund 2001). In traditional cultures, one of the applications of the rite of passage was as a means of ameliorating the consequences of disruptions to life course experiences. In many places in Central and Eastern Europe, those who did not enact the accepted sequence of life experiences became demons (Stomma 1981). In Slavic rites, there were analogies between wedding rituals and those observed at the funerals of young people, because the death of a young person without the enactment of the other events of the life cycle was considered particularly dangerous to the social community (Fischer 1921). There was the fear that those who had died a violent death and not been able to fulfil the natural sequence of their earthly life might prove especially vengeful, and so in the course of the funeral a symbolic marriage ceremony was held for that person (Barjatrović 1974; Kwaśniewicz 1974).

Interestingly, contemporary research reports also indicate that an upset to the defined order of life experiences can result in social underachievement: men who enter adult life in an unstructured fashion, i.e., in whose lives the order of the triad of finishing education, starting work, and marrying is inverted, earn less, have less prestigious professions, and are at greater statistical risk of divorce (George 1993). Other studies show that African Americans who have a less traditional sequence of life experiences—first work, then children, and only subsequently marriage—enjoy better health in later life (Jackson 2004). The study by Wrosh and Freund (Wrosh and Freund 2001) show that women who delay the decision to have children experience symptoms of depression when they reach the end of their fertility. Although there is nowadays greater freedom than in previous periods in terms of transitioning into successive stages of life (Ravanera et al. 2004), nonetheless, at the point of ‘biological transitions’ the potential risk of mental disintegration remains. One especially formative moment for the later life course is adolescence, because mental problems that surface in this period have a negative impact on future dedication to education, career, and income (George 2014). For instance, in connection with drug taking (Laub and Sampson 1993), there is the concept of the ‘gateway hypothesis,’ which suggests that use of marihuana in adolescence increases the risk of use of other illegal substances in adult life (Kandel 2002), although other key events can also influence the subsequent life course of such adolescents (Van Gundy and Rebellon 2010). Early adulthood is also a watershed point, because adverse experiences related to areas such as education can set in motion a chain of vocational and/or family misfortunes in a young person’s life that can lead to increased morbidity and mortality at a later age (Linda K. George 2014; Pearlin et al. 2005). Pregnancy and the post-partum period are also considered significant for the formation of the new identity, and this is also applicable to fathers (Draper 2003). Disruptions to mental health have a greater or lesser impact on the entire life course depending on the period of life in which they occur (Breslau et al. 2011). Another aspect of disruption to the chronological sequence of the life course is the protraction of or inability to progress out of a particular phase of an illness; an extended period of persistence of symptoms of an illness is a factor in its prognosis. In the case of Major Depressive Disorder, for instance, symptoms lasting longer than 24 months diminish the chances of a recovery (Bosworth et al. 2002; George 2014; Steffens et al. 2005). Family therapists also draw attention to the consequences of disruption of the family life cycle, stressing that issues from the initial phases of a new family’s life that are not addressed cause dysfunction in the family at later stages (Barnhill and Longo 1978; Combrinck-Graham 1985). They stress that remaining ‘frozen’ in a particular phase of the life cycle that does not correspond to socio-cultural norms leads to the emergence of psychopathological symptoms (Todd 1981). In such families, psychosomatic or psychotic problems can surface in one of its members (Combrinck-Graham 1985). The temporal and sequential perspective of the family life cycle also becomes clear in connection with loss in the family. An inability to balance one’s life is often related to a lack of closure or lack of acceptance of past losses, disappointments or conflicts, both with the dead and with the living (King and Wynne 2004). Losses in early family life are often linked to crises in later periods, as suggested by research into drug use, for instance. One such study shows that 87% of heroin addicts participating in it had experienced the premature loss or death of a significant family member, or their parents had experienced similar when they had been at the addicts’ own development stage (Coleman et al. 1986). One consequence of a watershed event in the life of a family such as the death of one of its members is the ‘pathological mourning’ syndrome, which can often bring the life of the entire family to a standstill (Walsh and McGoldrick 2004). In such cases, the frequent reaction is one of the excessive concentration—fixation—on the loss that has occurred (Boelen and Prigerson 2012), which disrupts or complicates progression of the individuals in the family into subsequent stages of development.

The concepts of rites of passage and the life course clearly illustrate the risk of disruption to the temporal sequence of progression of life experiences and of lingering in a particular stage of the life course or a certain phase of life crisis. The rite of passage concept introduces a structural model for resolving the problem of disrupted sequence of life experiences. The mechanism for this is to introduce a sequence of successive, but isolated phases of the change in progress and to finalize it in the form of engagement: transition to a new state.

### Liminality: Deconstruction, Integration, Transformation

The second of the processes within the matrix presented here refers in particular to the middle—liminal—phase of the transgression ritual, which symbolically illustrates the protracted state of being in a situation ‘without properties.’ This happens with the temporary suspension of a previous social or life role. The ritual symbolism of the liminal phase comprises opposing and conflicting content which symbolizes both the dying of the old and the birth of the new structure. It is a conceptualization of the type of crisis in which the prior life balance is fundamentally disrupted. Empirical studies relating to situations in which a previous identity is called into question reflect the situation of the individual who faces conflicting needs and demands. In life course theory, this is a point of risk connected with the irrevocability of certain decisions (Berger and Luckmann 1966), and is defined as a turning-point connected with a change to the previous life orientation (Rönkä et al. 2003) or a kind of correction to the previous life course (Clausen 1998). The term *transition* itself is directly linked with a shift into a new state, which is often symbolized by the ritual motif of migration, or the symbolic traveling of a road (Van Gennep 1960). The departure from ‘the ordered zone’ which is dramatized in many rites of passage is an immanent element of every change connected with leaving what is familiar and adapting to new, alien conditions. In the ritual presentation, this state that follows departure from the known and familiar is illustrated with symbols of anonymity, lack of ownership, and nakedness (Turner 1969). Other symbolic characteristics of this phase are chaos—suspended space, timelessness—time of celebration—liminal time, night, motionlessness, anti-matter, the unknown, death, incorporeality, ignorance, oblivion, and a lack of intentionality (Turner 1967, 1975; Van Gennep 1960).

Empirical studies into the experience of serious illness as a life-changing event largely reflect the structural and symbolic characteristics of the liminal phase. At such points, considerable deconstruction of the previous life balance is experienced, which causes the violation of prior assumptions about the self (Bury 1991), or the need to revise the individual biography (Rolland 1987). At this point, the constellation of ‘ego–body–angle on society’ calls for reconstruction (Bury 1982; Hyden 1997). If the illness is terminal, the previous life balance is disrupted entirely. In her analysis of the hospice as institution, Froggatt (Froggatt 1997) describes the disruption of order, chaos, and uncertainty occasioned by death and dying. The experiences of community and a certain equality—communitas—shared by those in a hospice and its personnel creates a kind of anti-structural order that helps to cope with the chaos and uncertainty caused by the suffering and threat.

A disrupted life balance generates many contradictions and conflicts. This finds symbolic form in the structure of the rite of passage, which at once forms a framework within which to integrate them. Focusing on the ritual representation of the convergence of opposites, individual–societal and biological–cultural, it characterizes the unity of the liminal, that which is ‘neither this nor that, and yet is both’ (Turner 1975). Bell sees the interaction between the individual and the societal within the ritual as a reflection of the tension between that which comes from nature and that which comes from culture. In her view, it is the integration of these opposites that forms the personality of the individual (Bell 1997). The intrusion of a biological factor, such as illness, as mentioned above, or the experience of puberty, aging, or pregnancy, elicits many conflicting emotions and creates the need for a new social definition of the individual’s situation. Empirical studies of people in situations of changing life roles or limbo confirm these characteristics. For instance, men experience their partners’ pregnancies in a highly ambivalent manner. They feel uneasy, in a state of limbo, often forced to deal with medical procedures that are incomprehensible to them (Draper 2003). The nature of this state of limbo is revealed by studies into the experience of motherhood or potential motherhood by women with cancer (Halliday et al. 2015). In such cases, diagnosis of the disease marks the beginning of a time of liminality; the familiar and predictable are destroyed and the new life space is filled with uncertainty, a vulnerability to injury, and unpredictability. Study participants emphasized that life-altering changes could take place in their lives at any moment. Even after their treatment was finished, they could not have any certainty with regard to the permanence of their health or to whether motherhood would be possible for them (Halliday et al. 2015). Enduring and integrating contradictory feelings are the way of overcoming this liminal position. Accommodating conflicting feelings and integrating them with one’s previous life history involve managing life crises, e.g., in people with co-dependency issues (Irvine 2000). Accommodating opposing tendencies within oneself is also the main force of ritual performances; transformation and overcoming crisis entail the integration of these conflicts and contradictions. The potential for change in the transition ritual is visualized by the balancing of symbols of decay with opposing symbols of growth and transformation: shelters and tunnels, which are both graves and wombs; lunar symbols; serpentine symbolism—snakes dying or shedding their old skin and reappearing in a new one; symbolism related to the bear, which dies in the autumn and is reborn in the spring; nakedness, which represents at once the baby and preparation for burial (Turner 1967, 1975; Van Gennep 1960). The potential for disintegration of the existing structure is visualized by the symbolic relinquishment of previously held societal positions in order to permit replanning. According to Turner (Turner 1967, 1969, 1986), liminal symbols may be linked with a state that may be described as ‘fruitful chaos,’ as a repository of many possibilities, a striving for new forms, the process of emergence of new structures. The self is created by the experience of overcoming successive developmental crises arising between what is biological and individual on the one hand and societal on the other (Erikson 1959). The three-level structure of the rite of passage is symbolically related to the division into stages of the conflict connected with the change in role: the separation phase is linked to the sadness at leaving the previous role, the middle phase represents confusion and lack of belonging, and the final phase may be understood as acceptance of the demands of the new role.

The theory of liminality illustrates the need to mediate between what requires the change involved in embarking on the next, new stage of life and that part of the self or its societal environment that is being abandoned.

The expression and regulation of the related emotions are illustrated most fully by the performative aspect of the ritual performance, which brings us to the third process in the model being examined here.

### Performativity: Regulation and Creation

In our attempt to answer the question of how a ritual performance can fit into the possibility for creating a new self, we shall first reference studies of the development of the infant self. This phenomenon is mediated in the multisensory interaction of the caregiver and the small child, where the process of mutual emotional regulation provides the framework for shaping the child’s self. Introducing this concept facilitates full comprehension of the third of the processes behind change and transformation isolated in this analysis, which is defined as ‘performativity.’ This process illustrates how the methods used in ritual performances regulate the emotions in order to survive the transformation of the self in their participants. The theory of intersubjectivity, which evolved from observation of babies (Beebe et al. 2003; Stern 1985, 2010; Trevarthen and Malloch 2000), describes the conditions in which the self is created and transformed from the beginning of life onward. It places emphasis on the intersubjective matrix embedded in the human mind, created in the process of the child’s interaction with its caregiver by the instigation of multisensory, multimodal interaction. A similar type of multimodal interaction takes place during the ritual performance, which is also the context for the formation of a new societal self for a given individual. In combination with eliciting and regulation of the emotions of the small child, interaction is the material from which the mind is formed (Beebe et al. 2003; Stern 1985, 2010). The central idea behind this conception is the need to experience the other as the foundation for experiencing the self and building one’s own ego. The self in this concept is fundamentally relational. The caregiver in his contacts with the child interprets her states and needs by engaging emotionally himself in contact with her, and then reflects them—which essentially means replaying them—in order for the child to be able to interpret her own experiences. This takes place in the course of repeated relational events which are steeped in emotions (Stern 2010). The theory of intersubjectivity emphasizes such interpersonal events as imitation (Melyzoff and Gopnik 1993), synchrony (Trevarthen and Malloch 2000), affect attunement (Beebe et al. 2003), intensity, abiding, and contours (Stern 2010). The concept of musicality that arises in this context refers to the harmonized movements and sounds between two people who express their motives by synchronizing their mutual behaviors (Stern 2010; Trevarthen 2008). According to Stern (2010), emotional fine-tuning is based on the linking and sharing of dynamic forms of vitality between various sensory modalities. In the description of vital communication, this refers to concepts including Trevarthen’s ‘communicative musicality’ (Trevarthen 2008), which draws attention to the communicative function of movements and sounds between two people who in this way express their motives and intentional states. We make mention of the developmental theory of intersubjectivity to show that humans are prepared in developmental terms—they have the intersubjective matrix—to experience the creation of their self in conditions of contact with another person, through the multimodal influence of various forms of vitality on their senses. In the situation of contact between child and caregiver, this involves multiple repetitions. We believe that the existence in the human mind of this intersubjective matrix instilled in infancy and based on the relational reflection of experienced states, emotional regulation, and multiple repetitions combined with the mobilization of a range of modalities: mimic, aural, kinetic expression along with its speed, rhythm, and emotional intensity form the mental basis for the impact of the performative aspects of ritual. McCauley and Lawson, in their study of the non-verbal message of the ritual, found that it is constructed in a way that symbolically appeals to the memory of its participants (McCauley and Lawson 2002). They posed a hypothesis on the existence of a ‘cognitive alarm,’ which moots that circumstances which cause emotional arousal stimulate the precise memory of the ritual by repeating and supplying plentiful associative material. This also facilitates the sustained focus of attention which is necessary to elicit emotional arousal. According to these scholars, the experience of a ritual rests on its eliciting of sensory and emotional arousal in the centers of memory and motivation. Sensory stimulation is highest when the individual undergoes a given ritual only once in her life, and when she harbors the conviction that divine intervention is one element of the ritual (McCauley and Lawson 2002). According to Turner, there is a structural relationship between the epistemic, emotional, and cognitive components of experience (Turner 1978). In his view, this is clearly visible in the sequential structure of the social drama. Combining the aesthetic and religious functions of the ritual, it becomes a performance that appeals to the shared senses but also to the sensory canals (Benari 1991). The regulation of both individual and social emotions is considered one of the major aims of ritual, in particular when its task is to deal with misfortune (Durkheim 1915) or to restore social order (Bajburin 1993; Toporow 1988). The drama of ritual accommodates both elements of arousing anxiety and ways of overcoming it. This aspect of ritual is referenced by scholars who seek in its structure construal of behaviors such as self-harming or para-suicidal actions (Hillman 1965; Peters 1994). Playing out practices of this nature places the individual in a situation of danger and contact with death, and thus reflects his inner tension (Peters 1994). Hillman believes that suicide attempts may be interpreted as a symbolic need to experience death in order to achieve radical change within the sphere of the ego (Hillman 1965). Ignorance and lack of awareness, illustrated in the ritual symbolism of nakedness or wandering, have a protective function in situations of turbulent change and transformation of human life. The successive stages of the ritual scenario, thanks to their clearly defined sequentially of events, help to regulate the emotions that are aroused. The performative aspect of the ritual performance is created in part by the presence of an audience. According to van Gennep, this is an expression of acceptance of those undergoing the ritual as members of the community, which at once illustrates the significant role of the existing social structure in charting the course of life experiences (Van Gennep 1960). Observers of the ritual experience its symbolic role in the context of their own life experiences (Benari 1991). Family therapists also reference the role of the audience in rites of passage (Beels 2007), because the change wrought over the course of the therapy is effected in the presence of, with the help of and in mutual interaction with close family members. These other family members present at the session also start to take a part in what the individual experiences in that time, and this provides a forum for both shared expression and transformation of the relations within the family, which is the objective of therapy (Beels 2007). Like the rite of passage, psychotherapy is also characterized by a kind of performativity, being outside the normal time of everyday life, addressing content that connects the past, the present, and the future, taking place in a specific territory and according to rules that differ from those of everyday life (Laird 1988), and arousing and regulating emotions.

In our attempt to underscore the role of the performative aspect of ritual performances in highlighting and regulating the experience of emotional states and in shaping the ego of the individual, we reference the existence in the mind of an intersubjective matrix based on bond and on multimodal processing of various sensory experiences. The drama of the rite of passage seems to fit into this intersubjective matrix, thus having the potential to influence the formation of the new self, not only symbolically but also directly. The arousal of strong emotions in the process of enacting the ritual involves an ambiguity surrounding the individual’s previous social position and role, and also the questioning of his previous self (Turner 1967; Van Gennep 1960). The performative aspects of the ritual performance, however, offer a framework for regulation of the emotions aroused by this manifestation of the ambiguity and conflictual character of transition periods. This is achieved by the process of bestowing meanings and multiple repetitions, which place the linear experience of the individual within the cyclical process, the sphere of the sacred (Hockey 2002; Levi-Strauss 1962). These processes of arousing emotion and subsequently regulating them and bestowing meanings on them create the possibility of transformation within the self. The symbolic expression of the experience of inner states and social messages in the process of intensive intersubjective interaction is a model for social and therapeutic impacts in life crisis situations.

## Conclusions

The theory of liminality and the life course theory form a conceptual matrix for the study and functional–structural understanding of change within the self as the individual crosses major developmental thresholds and experiences life crises. Analysis of these theories and studies based on them enabled us to isolate the dynamic mechanisms of life changes and transformations connected with the mutual relations between the individual and the social group, with especial emphasis on the following:

- The importance of preservation of the socially defined sequences of life experiences and drawing attention to the risk inherent in times of life crisis which take on chronic character;
- Recognition and normalization of temporary disintegration in the sphere of the self during a life crisis, leading to the co-existence of mutually contradictory mental content and inner conflicts that can later become integrated in the process of transformation or transition to a new state;
- Highlighting the performative character of the ritual performance as a symbolic structure regulating emotion and shaping the self, on the foundation of the developmentally inbuilt intersubjective matrix of mental development.

We thus wish to note that irrespective of whether we are analyzing a chronic illness, a crisis triggered by puberty, or an inability to close the mourning process, in each case we should reference the three processes listed above. First: preserving the sequence of life events reveals that each of these crises has the potential to disrupt subsequent stages of the life course, and each one will harbor the risk of chronic arrest in that particular phase of life. A given individual might never cast off the role of patient, might not accept the roles of adult life, or tackle them in a disrupted manner; he might remain to the end of his days in mourning and refuse all other social and life tasks. In each of these cases, a previously existing structure is destroyed and the individual moves into the second process characteristic for the liminal phase, that of the contradictions and ambiguities surrounding the role of the patient, torn between health and sickness, or even life and death; the role of the teenager, torn between childhood and adulthood; and that of the mourner, torn between the desire for physical contact with the deceased and the transformation of the bond with her into a mental bond. And finally comes the third process—performativity shows the importance in crisis situations of impacts which involve multimodal performative expression and social identification, e.g., participation in artistic and therapeutic groups or religious rituals, which involves arousing and at once regulating emotions.

Finally, we wish to stress that while the cultural scenarios of the life course change and evolve, their conflictual demands remain current. These include the desire for stability and security and yet the striving for change; biological and social relations; the risk of becoming mired in a transitory crisis situation, and the means of transmitting social support and social meanings. The concept of the rite of passage and the construction of the life course together supply a structural, sequential and processual framework for understanding and processing such challenges and dilemmas that is a main function of rites of passage and some contemporary social institution which try to deal with life crises.
